# Multiple tandem splicing silencer elements suppress aberrant splicing within the long exon 26 of the human Apolipoprotein B gene

**DOI:** 10.1186/1471-2199-14-5

**Published:** 2013-02-07

**Authors:** Umasuthan Srirangalingam, Scott A Akker, Dennis Norman, Naveenan Navaratnam, Shern L Chew, Bernard Khoo

**Affiliations:** 1 Department of Endocrinology, William Harvey Research Institute, Queen Mary University of London, John Vane Science Centre, Charterhouse Square, London, EC1M 6BQ, UK; 2 Current address: Argenta Discovery Ltd, 8/9 Spire Green Centre, Flex Meadow, Harlow, Essex, CM19 5TR, UK; 3 RNA Editing Group, MRC Clinical Sciences Centre, Division of Clinical Sciences, Imperial College London, Hammersmith Campus, Du Cane Road, London, W12 0NN, UK; 4 Current address: Department of Endocrinology, UCL Medical School, Royal Free Campus, Rowland Hill Street, London, NW3 2PF, UK

**Keywords:** Apolipoprotein B, RNA splicing, Splicing regulation, Splice sites, Splicing silencers

## Abstract

**Background:**

Apolipoprotein B (*APOB*) is an integral component of the chylomicron and the atherogenic lipoproteins LDL and Lp(a). Exon 26 of the *APOB* pre-mRNA is unusually long at 7,572 nt and is constitutively spliced. It is also subject to RNA editing in the intestine, which generates a shortened isoform, APOB48, assembled exclusively into chylomicrons. Due to its length, exon 26 contains multiple pseudo splice sites which are not spliced, but which conform to the degenerate splice site consensus.

**Results:**

We demonstrate that these pseudo splice sites are repressed by multiple, tandem splicing silencers distributed along the length of exon 26. The distribution of these elements appears to be heterogeneous, with a greater frequency in the middle 4,800 nt of the exon.

**Conclusion:**

Repression of these splice sites is key to maintaining the integrity of exon 26 during RNA splicing and therefore the correct expression of both isoforms of *APOB*.

## Background

Apolipoprotein B (*APOB*) is a component of the LDL, Lp(a) and chylomicron lipoprotein particles [1]. The full-length APOB100 isoform is mainly synthesized in the liver and is assembled into LDL and Lp(a). The pre-mRNA is subject to intestine-specific RNA editing by a complex known as the editosome, which includes a catalytic subunit, APOBEC-1, and several auxiliary factors. Editing occurs in exon 26 at position 6666 of the mRNA converting a C to a U, generating a premature termination codon (PTC), and producing a C-terminal truncated isoform, APOB48. This isoform is assembled into the chylomicron particle and is unable to bind the LDL receptor [2]. The constitutively spliced exon 26 of *APOB* is also unusual in that it is 7,572 nt long, far longer than the mean length of exons in the genome at 145 nt [3].

Pre-mRNA contains sequence elements defining the 5^′^ and 3^′^ ends of introns and the branch point. In mammals, the consensus sequences of these elements are highly degenerate [4]. As a result, matches to these consensus sequences are highly prevalent throughout the genome [5] and outnumber genuine splice sites by an order of magnitude in the human *HPRT* gene [6]. These pseudo splice sites are not normally employed in splicing. How the spliceosome is able to differentiate the few genuine splice sites from numerous pseudo splice sites is as yet unclear.

Other sequences within the pre-mRNA have been suggested to influence the choice between pseudo splice sites and genuine splice sites to allow accurate and reproducible splicing at the latter. Exonic and intronic splicing enhancer sequences (ESE and ISE, respectively) have been characterized, and in general these act by binding SR proteins which interact with the spliceosome to favour the use of particular splice sites [7-9]. Intronic and exonic splicing silencer sequences (ISS and ESS, respectively), which discourage the use of particular splice sites, have also been identified on functional and bioinformatic grounds [5,8,10]. Silencing elements bind silencing factors such as hnRNP A1 [11], polypyrimidine tract binding protein [12] and hnRNP H [13]. The balance between enhancing factors (e.g. SF2) and silencing factors (e.g. hnRNP A1) determines the selection of splice sites and alternative splicing [14,15].

Most of the pseudo splice sites in the human genome reside in introns, by virtue of their long lengths [5,6]. Long exons such as *APOB* exon 26, also contain many pseudo splice sites (*vide infra*). Correctly orientated pseudo 3^′^ and 5^′^ splice sites should trigger exon definition and erroneous splicing within the exon 26 sequence [16]. Alternatively, the genuine splice sites could be identified via a process of intron definition*.* This relies on the recognition of 5^′^ and 3^′^ splice site pairs over short introns <100 nt in *Drosophila*[17]. In human nuclear extracts and in HeLa cells, intron definition appears to operate below a threshold of 200–250 nt [18]. Intron definition, therefore, cannot easily explain the splicing of *APOB* exon 26 as the upstream intron Y and the downstream intron Z are too long at 506 and 403 nt respectively. *APOB* exon 26 provides a crucial test case for the hypothesis that the configuration of ESE and ESS within the pre-mRNA is important for the accurate and reproducible splicing of long exons.

## Results and discussion

### APOB exon 26 contains many pseudo splice sites

**Figure 1:**
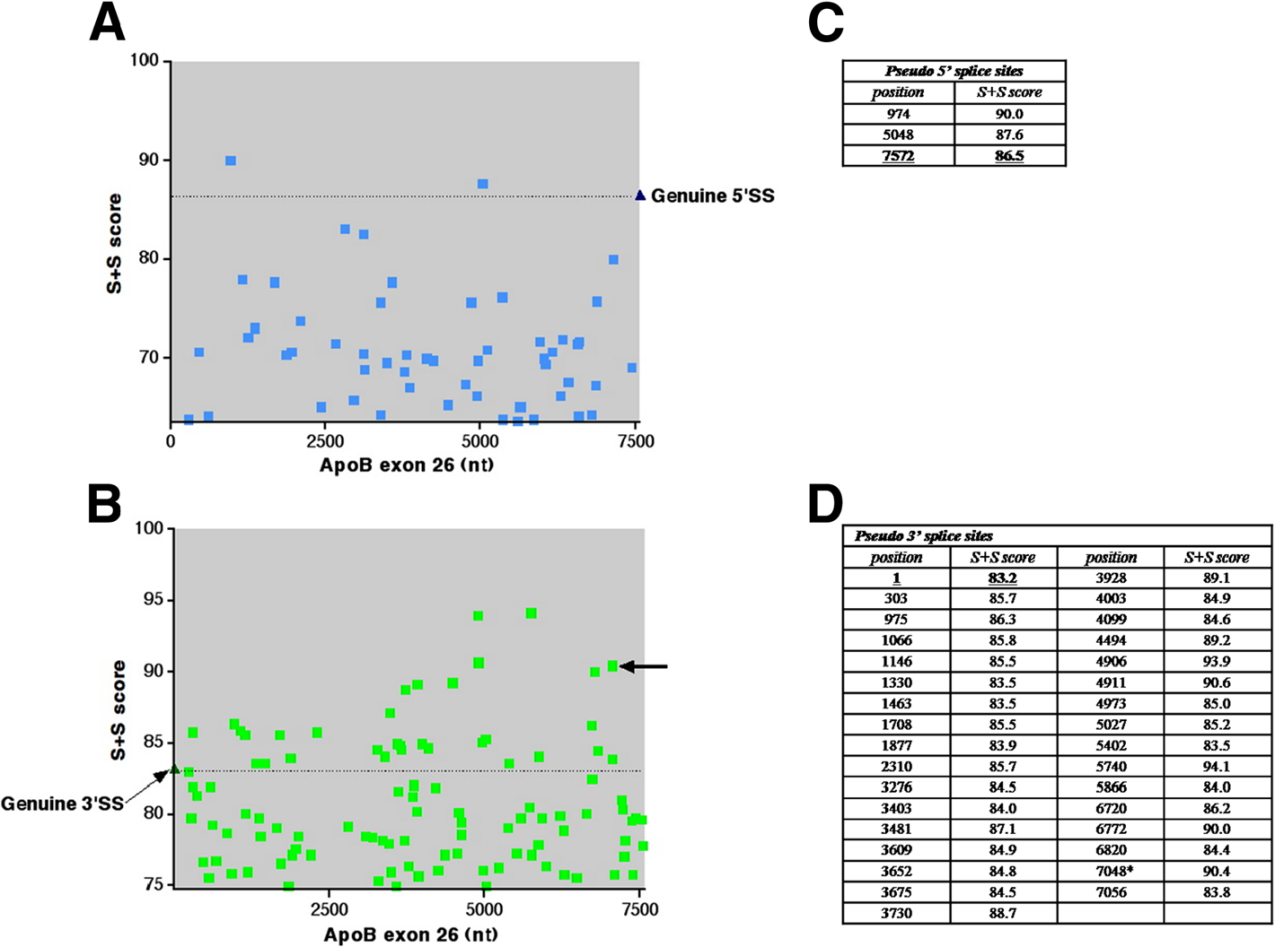
**Shapiro and Senapathy [**[4]**] scores for pseudo splice sites scoring higher than the lowest-scoring genuine *APOB *splice sites.** The dotted lines show the scores of the genuine splice sites flanking exon 26. (**A**) Scores of 5^′^ splice sites (ordinate) plotted against position in exon 26 (abscissa). (**B**) Scores of 3^′^ splice sites (ordinate) plotted against position of the in exon 26 (abscissa). The arrow indicates the pseudo splice site that is used in the alternative splicing event identified in Figure 6. (**C**) Table of positions and Shapiro and Senapathy scores of pseudo 5^′^ splice sites in exon 26 scoring higher than the native 5^′^ splice site at position 7572. (**D**) Table of positions and Shapiro and Senapathy scores of pseudo 3^′^ splice sites in exon 26 scoring higher than the native 3^′^ splice site at position 1. The asterisk indicates the pseudo splice site that is used in the alternative splicing event identified in Figure 6.

In order to assess the number of pseudo splice sites within *APOB* exon 26, we scanned the genomic sequence of *APOB* for matches to the 5^′^ and 3^′^ splice sites consensus sequences using the position score matrix of Shapiro and Senapathy [4]. A higher score out of 100 indicates a stronger splice site, and provides an estimate of the likelihood of a sequence being identified as an exon boundary and utilised in splicing. The genuine splice sites delineating the internal exons 2–28 were identified (Additional file 1: Table S1). The weakest 5^′^ splice site was that of exon 27 (score: 63.5) and the weakest 3^′^ splice site was that of exon 20 (score: 74.7). Using 63.5 and 74.7 respectively as the thresholds for the search, 54 5^′^ splice sites with scores ≥63.5 and 103 3^′^ splice sites with scores ≥74.7 were identified within the sequence of *APOB* exon 26 (Figure 1).

The 3^′^ splice site flanking exon 26 scored 83.2 and the 5^′^ splice site scored 86.5 (combined score 169.7). Using the exon 26 flanking scores of 83.2 and 86.5 as thresholds, 32 3^′^ splice sites and two 5^′^ splice sites within exon 26 were identified as closer matches to the consensus with higher scores than the genuine splice sites flanking exon 26. On theoretical grounds, therefore, each of these pseudo splice sites could be used in preference to the genuine splice sites, generating two possible 3^′^ shortened alternatives combining the 5^′^ pseudo splice sites with the 3^′^ splice site flanking exon 27, and 32 possible 5^′^ shortened alternatives combining the 3^′^ pseudo splice sites with the 5^′^ splice site flanking exon 25.

A search was also made for plausible pseudo-exons within the length of exon 26 using this subset of high-scoring pseudo splice sites. These were defined as sequences bounded by 3^′^ and 5^′^ pseudo splice sites in the right orientation, with lengths of 39–374 bp, these being the range of lengths for the other *APOB* internal exons. Using these criteria at least 3 alternative pseudo-exons could be identified that contain pseudo splice sites that are a better fit to the consensus than the genuine splice sites, and which on these bioinformatic criteria are more plausible exons than exon 26 itself.

Using an alternative method to identify splice sites, Maximum Entropy Modelling (see Additional file 2: Table S2) of the same region identified five 3^′^ splice sites and one alternative 5^′^ splice site within exon 26 which score higher than the native 3^′^ and 5^′^ splice sites [19]. Again using the same criteria, one strong pseudo-exon of 143 bp, with an appropriately positioned high scoring branch site was identified (position 4906–5048) [20]. Both these methods therefore identify plausible pseudo-exons which should in theory be spliced into the *APOB* mRNA instead of the actual exon 26.

### APOB exon 26 contains multiple silencing sequences along its length

**Figure 2:**
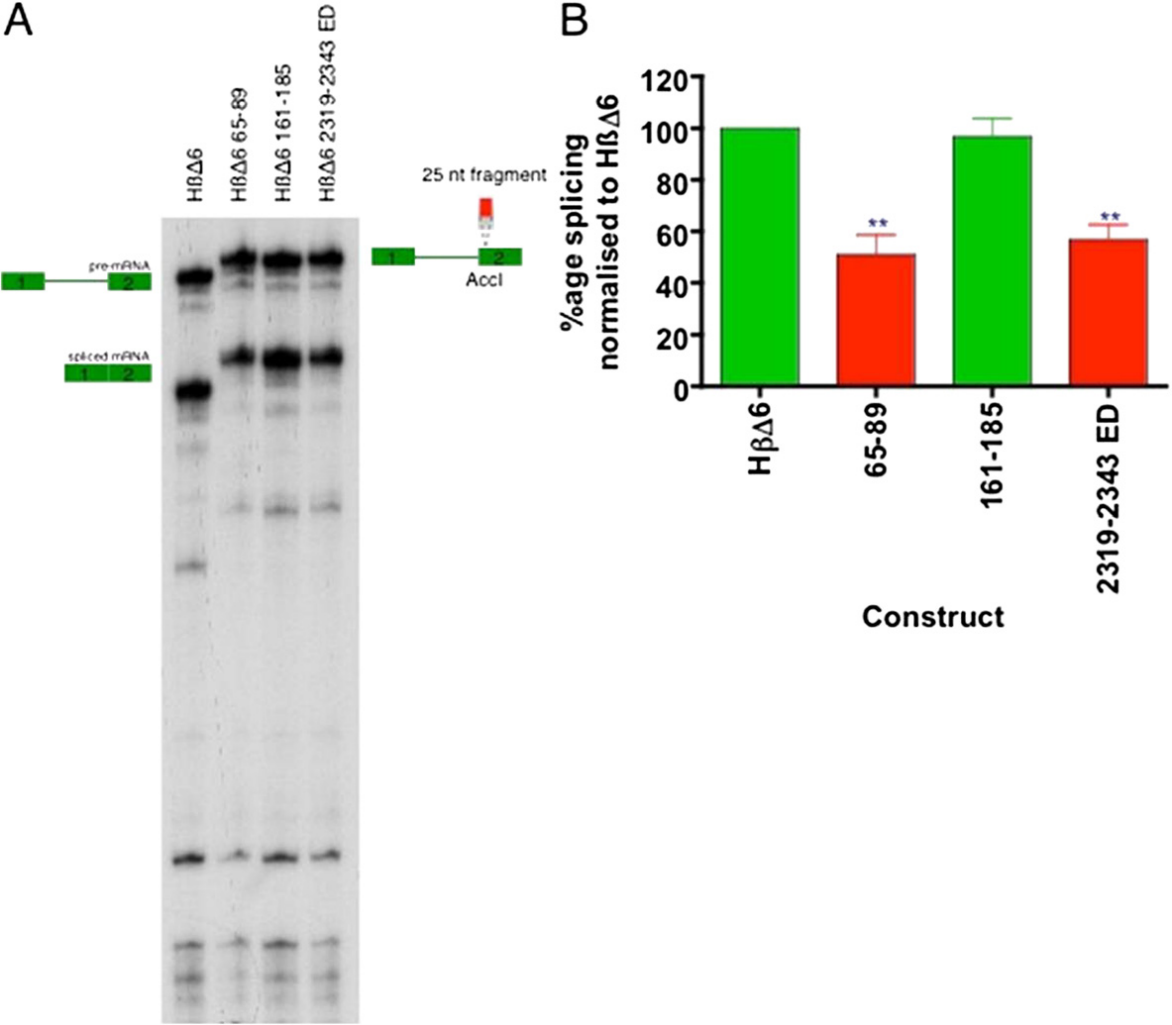
**Structure of the DNA ligase III splicing reporter and *in vitro *splicing assay of exon 26 sequences.** (**A**) *Top* Schematic showing the genomic structure of *APOB* exon 26 and adjacent exons. Boxes represent exons, the lines interconnecting exons represent introns. Numbers above exons and introns represent lengths of the respective elements in nucleotides. *E*xon 26 is divided into 400 nt fragments labelled 1 to 18. Note that fragment 19 is shorter than fragments 1–18 at 372 nt. *Bottom* shows fragment 5 in position at the 3^′^ end of the DNA ligase III splicing reporter, replacing the native ESS. (**B**) *In vitro* splicing of A constructs: 10% PAGE shown. *Lanes* M = pBR322-*MspI* digest marker; Cß = DNA ligase III C-ß construct without ESS; I3+4 = DNA ligase III C-ß with IGF-I exons 3 and 4 tagged to 3^′^ end; 1–19 = fragment 1 to 19 constructs. *Adjacent* cartoons show identity of bands. Note that the migration of the splicing intermediates of the DNA ligase III C-ß reporter is characteristically retarded on this high percentage gel. (**C**) Graph showing relative splicing efficiency of constructs, normalised to 100% = splicing efficiency of DNA ligase III C-ß construct without ESS.

We hypothesized that ESS might be responsible for silencing the pseudo splice sites, with two possible architectures for ESS in exon 26. Firstly, a single or a few strong ESS sequences might exist which are able to influence large areas of exon 26, similar to hnRNP A1. This is able to bind to high affinity sites, cooperatively bind, propagate along an exon, and antagonize SR protein function [21]. In this context it should be noted that exon 26 contains sequences that resemble consensus high-affinity hnRNP A1 sites [22] such as 2666–2671 (UAGAGU – found in construct 7 of Figure 2B) and 4114–4119 (UAGGGA – found in construct 11 of Figure 2B). Secondly, exon 26 might contain multiple ESS distributed along the length of the exon.

The length of exon 26 makes it difficult to test for the existence of ESE and ESS in exon 26 in their native context. Therefore, we turned to a heterologous splicing reporter, DNA ligase III. This two-exon construct contains a C exon and an alternatively spliced ß exon which is controlled by an ESS within the ß exon. Deletion of the ESS allows C to splice to ß efficiently *in vitro*[23]. Candidate sequences derived from exon 26 were therefore cloned at the 3^′^ end of the ß exon, replacing the native ESS, and these constructs were transcribed and tested for splicing *in vitro* (Figure 2A). Suppression of C-ß splicing indicates presence of ESS activity within the candidate sequence.

Figure 2B shows the results of testing non-overlapping 400 nt fragments derived from exon 26 in this system. DNA ligase III C-ß splices efficiently, as does a control construct containing 339 nt of IGF-I exons 3 and 4, tagged to the 3^′^ end of DNA ligase III C-ß (see lanes Cß and I3+4). However, tagging 376–400 nt fragments of exon 26 causes silencing of splicing of the C-ß intron (lanes 1–19), with variable silencing activity – compare lanes 1–4, 17, 18 with lanes 5–16, 19. Silencing activity appears to be strongest in the middle 4800 nt of sequence corresponding to fragments 5–16.

We conclude, therefore, that the sequence of exon 26 contains splicing silencing activity throughout its length, but that this activity is heterogeneously distributed. There is no evidence of a single super-ESS in exon 26, for example centered around the potential high-affinity hnRNP A1 sites.

### Microarchitecture of ESS in exon 26

In order to further investigate the distribution of ESS in exon 26, overlapping 25 nt sequences derived from fragment 1 and from fragment 6 were tested using the DNA ligase III *in vitro* splicing system. We selected fragment 1 and fragment 6 as exemplars of relatively well-spliced and relatively silenced fragments respectively. Moreover, as fragment 6 contains the RNA editing site, we wished to see if silencing activity was related to the RNA editing activity.

**Figure 3:**
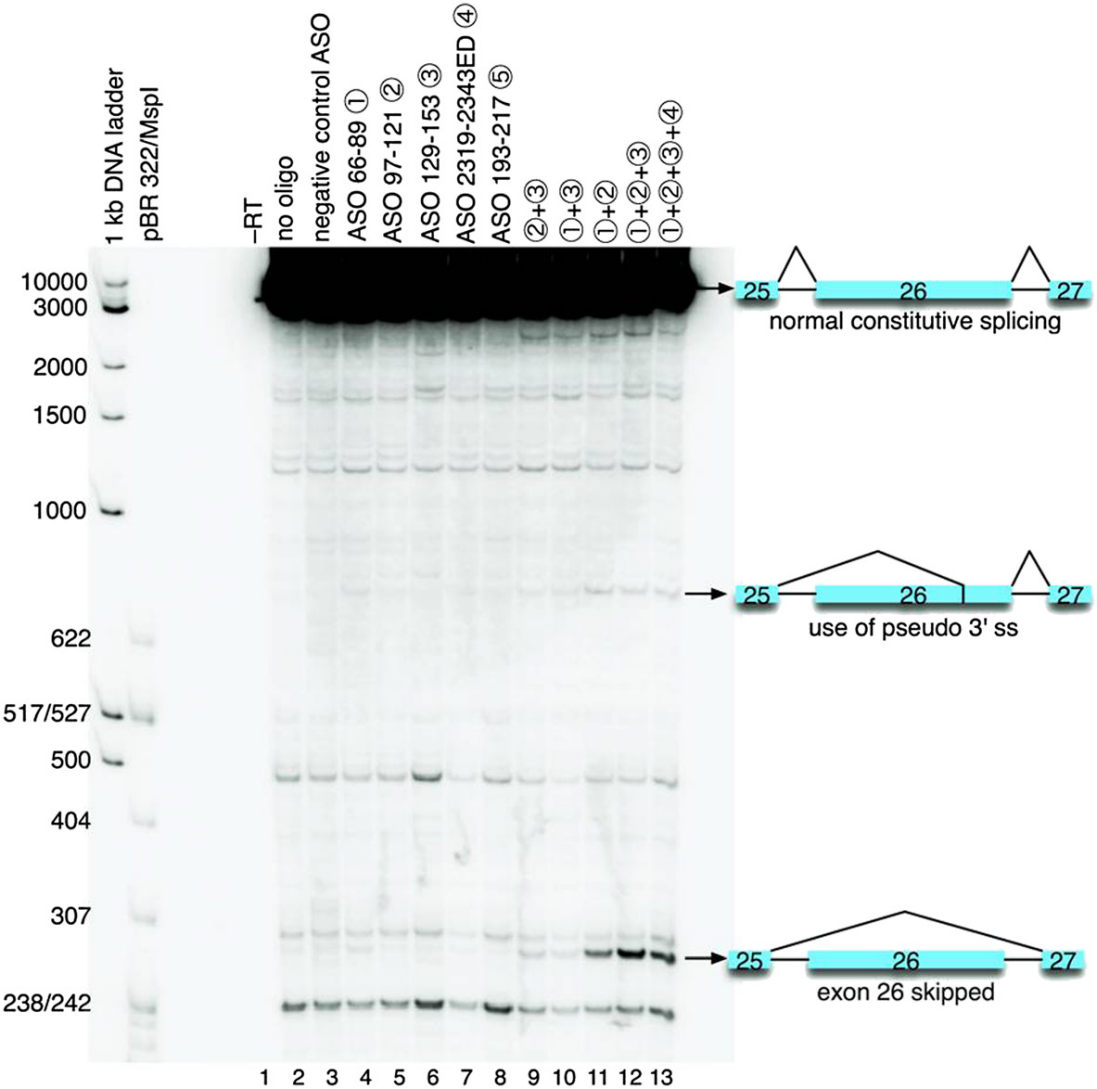
**Microarchitecture of ESS activity in exon 26 sequences derived from fragment 1.** (**A**) *In vitro* splicing assay of DNA ligase III C-ß splicing reporter, tagged with 25-mers derived from fragment 1 (1 hour splicing at 30°C). 10% PAGE shown. *Lanes* Cß = DNA ligase III C-ß construct without ESS; Cß ex27 1–25 = Cß + nt 1–25 of exon 27; Cß REP = Cß + native ESS; Cß IGF-I ex3 1–25 = Cß + nt 1–25 of exon 3 from IGF-I; DL3 Cß ex 26 lanes = nts of exon 26 shown. *Adjacent* cartoons show identity of bands. (**B**) Quantification of splicing efficiency, normalised to DNA ligase III C-ß reporter. Bars show splicing efficiency from n=4 experiments with DNA ligase III C-ß = 100%. Error bars show S.E.M. Overall one-way ANOVA shows p <0.0001. Comparison to DNA ligase III C-ß for each construct by Bonferroni’s multiple comparison test: statistically significant differences from efficiency of DNA ligase III C-ß splicing shown by *= p <0.01, **= p <0.001.

**Figure 4:**
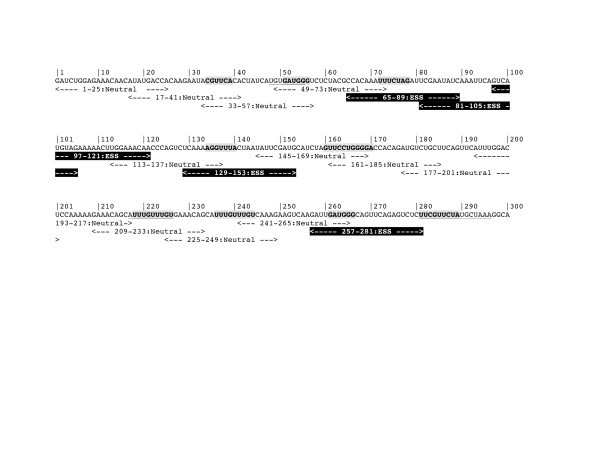
**Microarchitecture of ESS activity in exon 26 sequences derived from fragment 6.** (**A**) *In vitro* splicing assay of DNA ligase III C-ß splicing reporter, tagged with 25-mers derived from fragment 6 (1 hour splicing at 30°C). 10% PAGE shown. *Lanes* Cß = DNA ligase III C-ß construct without ESS; Cß ex27 1–25 = Cß + nt 1–25 of exon 27; Cß REP = Cß + native ESS; Cß IGF-I ex3 1–25 = Cß + nt 1–25 of exon 3 from IGF-I; DL3 Cß ex 26 lanes = nts of exon 26 tested are shown above each lane; lane 2319–2343 encompasses the RNA editing site and mooring sequence (ED); ED sequence mutations T9A, T9C, A11C, G15C, A19T shown in right-hand five lanes. *Adjacent* cartoons show identity of bands. (**B**) Quantification of splicing efficiency, normalised to DNA ligase III C-ß reporter. Bars show splicing efficiency from n=4 experiments with DNA ligase III C-ß = 100%. Error bars show S.E.M. Overall one-way ANOVA shows p <0.0001. Comparison to DNA ligase III C-ß for each construct by Bonferroni’s multiple comparison test: statistically significant differences from efficiency of DNA ligase III C-ß splicing shown by *= p <0.01, **= p <0.001.

Figure 3A shows the result of testing these constructs, made with sequences derived from fragment 1. At this resolution, there is a heterogeneous distribution of ESS activity, with certain sequences such as 65–89 showing silencing better than the native ESS of DNA ligase III, REP and other sequences such as 113–137 showing no significant effects on splicing (Figure 3B). Identically spliced mRNAs appear as different sized bands in the lower parts of Figures 3A and 4A. This is likely to represent variable degrees of exonuclease degradation to which mRNAs are susceptible in the nuclear extract used for the assay.

Figure 4 shows the result of testing constructs made with sequences derived from fragment 6. In contrast to the fragment 1 sequences, the fragment 6 sequences show less heterogeneity with most sequences tested showing ESS activity (see Figure 4A and 4B). We conclude, therefore, that exon 26 contains multiple ESS sequences along its length in a heterogeneous distribution. More frequent silencing activity is seen with the 25-mers derived from fragment 6 than fragment 1, consistent with the greater silencing activity of fragment 6 compared to fragment 1.

### ESS identified by the DNA ligase III C-ß reporter also silence the HßΔ6 reporter

**Figure 5:**
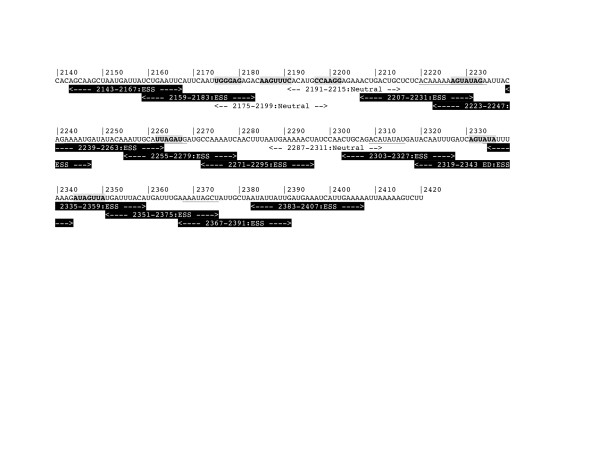
**Activity of key ESS sequences in the context of HßΔ6.** (**A**) The indicated sequences from exon 26 were cloned into the AccI site of exon 2 and tested for *in vitro* splicing activity (2 hour incubation, 30°C). 5% PAGE shown. *Adjacent* cartoons show identity of bands. (**B**) Quantification of splicing efficiency, normalised to HßΔ6 reporter. The bars show splicing efficiency, normalised to HßΔ6 for n=9 experiments, error bars show S.E.M. Overall one-way ANOVA shows p <0.0001. Comparison to HßΔ6 splicing efficiency for each construct by Bonferroni’s multiple comparison test: statistically significant differences shown by **= p <0.001.

In order to ensure that the ESS activity was not dependent on the splicing reporter, key sequences were also tested in the ß-globin splicing reporter HßΔ6 [23,24]. In this different context, the 25-mers 65–89 and 2319–2343 ED were also able to repress HßΔ6 splicing by ~50%, whereas the neutral sequence 161–185 did not significantly repress splicing (Figure 5).

### The sequence requirements of RNA editing are distinct from ESS activity

RNA editing of *APOB* is dependent on an 11 nt mooring sequence 3^′^ to the RNA editing site itself, which represents a binding site for the editosome [25]. The sequence spanning the RNA editing site and mooring sequence (ED) shows ESS activity (see Figure 4A, lane 2319–2343 ED). As RNA editing and splicing are linked [26] and as both processes may share common components, such as KSRP [27] and CUGBP2 [28], we wondered whether the silencing activity of ED might be related to its ability to support RNA editing. To this end, several point mutations were constructed in ED, three of which are known to knock out RNA editing (T9A, T9C, A11C), one of which is neutral with respect to RNA editing (G15C), and one of which is intermediate in reducing RNA editing activity (A19T) [28]. These constructs were tested for splicing activity (see Figure 4A, right-hand 5 lanes). The mutations did not affect the silencing activity of ED, suggesting that the sequence requirements of RNA editing are distinct from ESS activity and that perhaps different factors are responsible for silencing activity vis-à-vis RNA editing.

### Antisense oligonucleotide blockade of ESS causes alternative splicing of APOB

**Figure 6:**
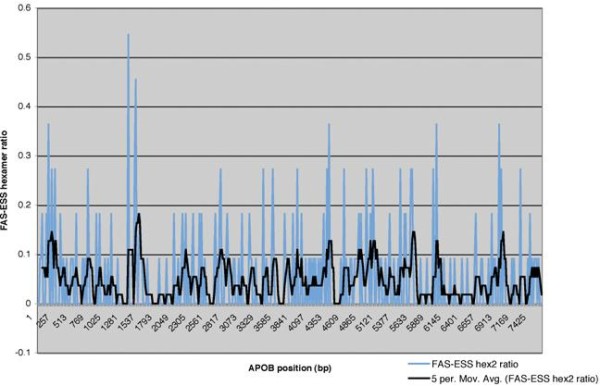
**Antisense oligonucleotides directed against identified ESS elements cause alternative splicing of APOB in HepG2 cells.** In lanes 1 and 2 the cells were not transfected with ASOs. The –RT control PCR (lane 1) was performed without reverse transcriptase. In lane 3, the cells were transfected with negative control ASO at 500 nM. Lanes 4–13 were transfected with the indicated ASOs at 125 nM each; the total concentration of ASO was adjusted to 500 nM with negative control ASO. Cells were incubated for 48 hours and RT-PCR was carried out on the total RNA extracted from these cells with oligonucleotides annealing to the adjacent exons 25 and 27. The positions of the bands corresponding to the *APOB* mRNA species with constitutive exon 26 inclusion (top), pseudo 3^′^ splice site activation (middle) and skipping of exon 26 (bottom) are indicated on the right side. Fragment lengths in bp of the markers are indicated on the left side.

To investigate the role of these ESS elements *in vivo*, we turned to 2^′^-*O*-methyl antisense oligonucleotides (ASOs), which can direct alternative splicing by binding to splice sites or regulatory elements [29,30]. These ASOs were targeted at identified ESS, namely elements 66–89, 97–121, 129–153, 2319–2343 ED (Figure 6: labelled - respectively). We also made an ASO targeted to a neutral region, 193–217 (labelled), and a negative control ASO which did not match any sequences within *APOB*. These ASOs were transfected into HepG2 cells, which synthesize and secrete APOB. The splicing of *APOB* exon 26 was assessed by RT-PCR [30].

As shown in Figure 6, targeting ASOs individually to each ESS did not cause significant alternative splicing (compare lanes 2 and 3 with lanes 4–7). Targeting the neutral sequence 193–217 did not alter splicing (compare lanes 2 and 3 with lane 8). Targeting another neutral sequence 2191–2215 did not alter splicing (Additional file 3: Figure S1: compare lane 6 with lane 2). However, when mixtures of ASOs were combined, especially when ESS 66–89, 97–121 and 129–153 were targeted simultaneously, we saw significant alternative splicing of *APOB* to two alternative isoforms (compare lanes 2 and 3 with lanes 9–13): one in which exon 26 was skipped to join exons 25 and 27 together; and one in which a pseudo 3^′^ splice site (Shapiro and Senapathy score 90.4 and Maximum Entropy Modelling score 10.04) was selected at position 7048 within exon 26 (see also Figure 1B – this splice site is indicated with the arrow). Maximum Entropy Modelling ranks this pseudo 3^′^ site as the highest scoring and strongest 3^′^ site within APOB exon 26 (Additional file 2: Table S2), in comparison to the native 3^′^ splice site (score 7.68). This therefore implies that: (1) these elements have a function in suppressing pseudo splice site selection *in vivo*; and (2) that these elements have functional redundancy.

While the splice site strength of this cryptically activated 3^′^ splice site is higher than the native 3^′^ splice site it is unclear why a group of ESS approximately 7,000 nt upstream might be involved in repressing this pseudo splice site. We may speculate that disruption of exonic silencers allows more efficient aggregation of splicing factors around cryptic splice sites further downstream, thereby increasing the kinetics of spliceosome assembly and allowing minor isoforms to become apparent. The other possibility is that interference with ESS allows silencing protein activity to become more influential at the constitutive 3^′^ splice site. Other methodological explanations include the possibility that pseudo splice sites nearer the ESS sites are selected which would give significantly larger products which were not amplified efficiently by the PCR in comparison to the shorter transcripts, or that could not be distinguished from constitutively spliced transcript by gel electrophoresis under the conditions which we used.

### Computational identification of ESS is less specific and sensitive than splicing assays

Experimental and computational models, such as FAS-ESS hexamers and putative ESS (PESS) octamers as defined by Zhang and Chasin [8], can be used to predict ESS sites in exon 26 and which may then be compared with sites identified in our assay. FAS-ESS hexamers appear to occur along the exon 26 sequence (Additional file 4: Figure S2), but there does not appear to be any bias towards more frequent appearances within the middle of the exon as noted in our assay. We found that there was a variable correlation of FAS-ESS hexamers within 25mer ESS identified in our assays (Additional file 5: Figure S3 and Additional file 6: Figure S4) but overall these appear to be less sensitive (50%) and less specific (40%) than the *in vitro* splicing assay. A similar picture was again apparent with the PESS matches (Additional file 5: Figure S3 and Additional file 6: Figure S4).

It has also been suggested that the distribution of splicing enhancer sequences can influence the use of genuine splice sites over pseudo splice sites. We therefore searched for ESE sequences within exon 26, using tools derived from computational and experimental modeling [7,8,31-33] to see if there might be such a bias in the distribution of ESE. However, putative ESE were noted all along the length of the exon and do not appear to have a bias for the ends of the exons, in keeping with other similar studies [34], suggesting that ESE do not appear to contribute to the selection of genuine splice sites in exon 26. This is further confirmed by the fact that we did not detect any significant enhancement of reporter splicing by the tested sequences.

### Secondary structure

A prediction of the secondary structure folding of APOB exon 26 pre-mRNA and its flanking introns, utilising the mfold web server (see Additional file 7: Table S3) shows that Region A6, which has inherently higher silencing activity compared with region A1, is predicted to contain more secondary structure [35]. In particular, the ESS containing the editing site, targeted by ASO (2319–2343), contains a high degree of secondary structure (76% of the sequence predicted to be base-paired). Work by Hiller and colleagues [36] suggests that splicing elements are more effective when they are in single-stranded conformations, which does not fit with our observation that the A6 region (with the highest density of ESSs) is predicted to form strong secondary structures, and more so than the A1 region which has a lower density of ESSs. Moreover, as the formation of secondary structure by a particular sequence is highly dependent on the context of its neighbouring sequences, it is unclear how the ESSs, if they are working primarily by means of mechanisms dependent on the formation of secondary structure, could inhibit the splicing of the two different heterologous splicing reporters used here. For these reasons we believe that secondary structure may not be a key influence in pseudo splice site silencing in exon 26.

### Nonsense-mediated decay

In addition to regulation of splicing, other mechanisms have been proposed to ensure that mature mRNAs have been accurately spliced. Nonsense-mediated decay (NMD) is conceivably a mechanism for suppressing PTC-containing mRNAs generated by aberrant splicing to cryptic splice sites, and is in some cases a mechanism for regulation of protein expression [37]. However, despite the fact that RNA editing generates a PTC in the middle of exon 26, edited *APOB* mRNA appears to not be subject to NMD, nor is a PTC-containing *APOB* mRNA isoform skipping exon 27 [30]. In the case of exon 26, the binding of the APOBEC-1 complementation factor (ACF), a component of the editosome, is important in protecting the edited RNA from NMD [38]. Further, out of the 37 alternative isoforms generated by use of high-scoring pseudo splice sites in exon 26, 16 are predicted to splice in-frame and do not contain PTCs. Therefore, NMD is not a plausible mechanism for policing the selection of splice sites in *APOB* splicing.

## Conclusions

The splicing of RNA requires the accurate selection of genuine splice sites over numerous pseudo splice sites, and this is facilitated by additional RNA sequence information in the form of splicing enhancers and silencers. Accurate splicing of exon 26 is necessary to ensure that APOB is expressed correctly and assembled into the correct lipoprotein particles.

To our knowledge, this is the first time a partial survey of splicing elements has been carried out in a long exon. In most experimentally characterised cases, ESS have been isolated as single elements or sometimes bipartite elements of variable length from 5 nt [39] to 119 nt [40], usually in the context of alternative splicing. In exon 26, our data demonstrate that there exist multiple, tandem ESS, a unique configuration of ESS. We show, *in vivo*, that blocking selected ESS with antisense oligonucleotides triggers the selection of the strongest 3^′^ pseudo splice site within exon 26, i.e. cryptic activation of the pseudo splice site. Although constitutive splicing of exon 26 at the native splice sites continues to generate the dominant isoform, and the alternative splicing effects are small, we speculate that this may be ascribed to poor access of blocking oligonucleotides to the spliceosome and the fact that we have only targeted a small number of multiply redundant ESS. This latter notion is supported by the fact that blockade is only effective with combinations of ASOs.

We hypothesize that these multiple ESS may serve to suppress the pseudo splice sites within it, and we propose that similar mechanisms may govern the accurate splicing of other long exons in the genome.

Furthermore, it is possible that polymorphisms within the exon 26 sequence, even if translationally silent, may perturb these silencers and disturb splicing of exon 26, leading to subtle alterations of APOB expression and function which may underlie inter-individual differences in lipoprotein levels and function [41,42].

## Methods

### Computer analysis of sequences

The *Homo sapiens* genomic DNA sequence of APOB was obtained from Ensembl (HUGO ID: APOB) [43]. The SpliceSiteFinder server was used to scan exon 26 for potential splice sites, scored according to the matrices of Shapiro and Senapathy [4,44]. ESEfinder was used to scan for potential SR protein binding sites [33,45]. The RESCUE-ESE web server was used to scan for putative ESE hexamers [9,46]. The FAS-ESS web server was used to scan for putative ESS hexamers [10,47]. The PESx web server was used to scan for putative ESS as defined by Zhang and Chasin [8,48]. Maximum Entropy Modelling [19] was additionally used to scan exon 26 for potential splice sites.

### Construction of DNA ligase III constructs

All oligonucleotides were obtained from Sigma-Genosys Ltd., Cambridge, UK. 400 nt fragments of exon 26 were amplified from human genomic DNA or HepG2 cDNA, and coupled to T7 DNA ligase III C-ß DNA template [49] by overlap-extension PCR [50] employing Phusion DNA polymerase (Finnzymes OY, Finland). Sequences were confirmed by direct DNA sequencing. T7 DNA ligase III constructs containing 25-mer sequences from exon 26 were constructed by PCR employing reverse oligonucleotides containing the reverse-complement of the desired sequence appended to the 5^′^ end of the DNA ligase III C-ß sequence. HßΔ6 constructs were made by annealing complementary oligonucleotides containing the desired 25-nucleotide sequences with 5^′^-CT and 5^′^-AG overhangs, and these were cloned into the AccI site within the second exon of HßΔ6 [23,24]. Oligonucleotide sequences are available on request.

### In vitro analysis of splicing

Labelled, capped RNA transcripts were generated using the appropriate RNA polymerase (Ambion, Inc., Huntingdon, UK), 1 mM 7mG(ppp)G cap structure analogue (NEB, Hitchin, UK), 0.5 mM each of ATP, UTP, CTP and 0.05 mM of GTP, and 5–10 μCi [α-^32^P] GTP (Perkin-Elmer LAS Ltd., Beaconsfield, UK) [51]. 20 fmol of labelled RNA was employed in *in vitro* splicing reactions with 10 μl of HeLa nuclear extract (Computer Cell Culture, Mons, Belgium) for 1 hour at 30°C (DNA ligase III constructs), 2 hours at 30°C (HßΔ6 constructs) or on ice as indicated [52]. Purified spliced RNA was analysed with 5% or 10% urea-PAGE (Sequagel, National Diagnostics, Hessle, UK) and autoradiography. Quantification of autoradiographs was performed using FujiFilm Image Gauge software version 4.2 (FujiFilm Medical Systems, Stamford, CT, USA).

### Antisense oligonucleotide transfection of HepG2 cells

HepG2 cells were maintained in Minimum Essential Medium with Earle’s salts and glutamine, 10% fetal bovine serum (Gibco/Invitrogen, Paisley, UK) at 37°C under 5% CO_2_. 2^′^-*O*-methyl ASOs were ordered from Dharmacon (Lafayette, CO, USA). The sequences (5^′^-3^′^) of the ASOs were as follows: ASO 65–89 GAUAUUCGAAUCUAGAAAUUUGUGG; ASO 97–121 UUGUUUCCAAGUUUUUCUACAUGAC; ASO 129–153 CAUCGAAUAUUAGUAAACCUUUUGA; ASO 2319–2343 CUUUAAAUAUACUGAUCAAAUUGUA; ASO 93–217 UGCUGUUUCUUUUUGGAGUCCAAAU; negative control ASO GGCCGAUCCGUCAGUCCA.

ASOs were transfected in 12-well plates, with 1 × 10^6^ cells per well, using 2.4 μl Lipofectamine 2000 in 200 μl OptiMEM I (Invitrogen) as per the manufacturer’s protocol, then incubated for 48 hours before harvesting. Total RNA was isolated from cells using the RNeasy Micro kit (Qiagen, Crawley, UK). The RNA concentration was quantified using the NanoDrop 1000 (Thermo Scientific, Waltham, MA, USA), and 2.5 μg was reverse-transcribed using Transcriptor reverse transcriptase (Roche Applied Science, Burgess Hill, UK) with an oligo dT_15_ primer as per the manufacturer’s protocol. 0.25 μl of the cDNA was then analysed in an RT-PCR reaction with Exl DNA polymerase (Stratagene, La Jolla, CA, USA) and 0.5 μCi [α-^32^P]-dCTP (Amersham, Little Chalfont, UK) using the following primers: forward (exon 25) GCCATCTCGAGAGTTCCAAG and reverse (exon 27) GTCACGGTGTGCAAATGTTC according to the manufacturer’s instructions. The PCR reaction was then subjected to PAGE on a non-denaturing 6% gel, and autoradiographed. The identity of each PCR product of interest was confirmed by excision and sequencing.

### Statistical analysis

Statistical analysis was performed using Prism version 4.0c (GraphPad Software, Inc., San Diego, CA, USA). Comparisons between quantitated datasets were performed using one-way ANOVA. Bonferroni’s test was used to analyse the differences in splicing efficiency between individual constructs and control constructs.

## Competing interests

B.K. is an inventor on a patent relating to therapeutic uses of alternative splicing in *APOB*. The authors declare that they have no other competing interests.

## Authors’ contributions

BK performed the experiments, computational and statistical analyses, and is the primary author of the manuscript. U.S. performed the antisense oligonucleotide experiment. SAA provided reagents and practical help with experiments and analysis. DN performed preliminary experiments. NN designed the initial experiments. SLC conceived the project and designed the initial experiments. All authors read and approved the final manuscript.

## Supplementary Material

Additional file 1: Table S1 Shapiro and Senapathy [4] scores for the splice sites flanking the internal exons 2–28 of *APOB*. Click here for file

Additional file 2: Table S2 Maximum Entropy Modelling scores [19] for 3^′^ and 5’ splice sites. Only motifs with higher scores than the native splice sites (italics) shown. Branch point sequences motifs with appropriate strength (score >70) and positioned within 50 nucleotides of a 3^′^ splice site capable of generating a pseudoexon [20]. Click here for file

Additional file 3: Figure S1 Antisense oligonucleotides directed against identified ESS elements of *APOB* exon 26 in HepG2 cells – additional control oligonucleotide included. In lanes 1 and 2 the cells were not transfected with ASOs. The –RT control PCR (lane 1) was performed without reverse transcriptase. Lanes 3–6 were transfected with the indicated ASOs at 250 nM each. An additional control oligonucleotide targeting a neutral sequence is shown in lane 6. Cells were incubated for 48 hours and RT-PCR was carried out on the total RNA extracted from these cells with oligonucleotides annealing to the adjacent exons 25 and 27. The positions of the bands corresponding to the *APOB* mRNA species with constitutive exon 26 inclusion (top), pseudo 3^′^ splice site activation (middle) and skipping of exon 26 (bottom) are indicated on the right side. Fragment lengths in bp of the markers are indicated on the left side. Click here for file

Additional file 4: Figure S2 Frequency of FAS-ESS hex2 hexamers in the APOB exon 26 sequence. FAS-ESS hex2 subset refers to those hexamers found at least twice within recovered decamers from the FAS-ESS procedure [47]. Hexamers were counted in a non-overlapping 16 bp windows and normalised to the number of possible hexamers per 16 nt. Blue line shows normalised hexamer ratio plotted for each window. Black line shows a moving average over 5 windows. Click here for file

Additional file 5: Figure S3 Computational identification of potential ESS sequences within the exon 26 sequence. The exon 26 sequence is shown in black, with numbers above denoting the position in exon 26. 25-mers tested in the DNA ligase III reporter system are shown below the exon 26 sequence, sequences with ESS activity are denoted by white-on-black text, neutral sequences are denoted by black-on-white text. Sequences containing hexamers identified by the FAS-ESS server program are in bold [47]. Matches to the PESS sequences identified by Zhang and Chasin [8] are underlined. Click here for file

Additional file 6: Figure S4 Computational identification of potential ESS sequences within the exon 26 sequence derived from fragment 6. The exon 26 sequence is shown in black, with numbers above denoting the position in exon 26. 25-mers tested in the DNA ligase III reporter system are shown below the exon 26 sequence, sequences with ESS activity are denoted by white-on-black text, neutral sequences are denoted by black-on-white text. Sequences containing hexamers identified by the FAS-ESS server program are in bold [47]. Matches to the PESS sequences identified by Zhang and Chasin [8] are underlined. Click here for file

Additional file 7: Table S3 Degree of base-pairing secondary structure predicted for regions of *APOB* exon 26 using the Mfold web server [35]. These include the native 3^′^ and 5^′^ splice sites, parts of region A1 (1–281) and A6 (2143–2407) investigated *in vitro* (see Figures 2 & 3) and the regions of exon 26 targeted by the ASOs 1–6. Secondary structure was analysed using the whole of exon 26 in addition to the entire length of the adjacent introns either side. Figures shown are the number of nucleotides predicted to be base-paired using the 4 most stable structures predicted (range of ΔG −2245.70 to −2244.2 kcal/mol) Final column gives the average number of nucleotides base-paired and the %nucleotides base-paired for the given sequences. Click here for file
