# Supplementary material for: Multiple tandem splicing silencer elements suppress aberrant splicing within the long exon 26 of the human Apolipoprotein B gene
Source: BMC Mol Biol. 2013 Feb 7;14:5. doi: 10.1186/1471-2199-14-5 (PMC3640928; doi:10.1186/1471-2199-14-5)
Supplement: Additional file 1: Table S1 — Shapiro and Senapathy [4] scores for the splice sites flanking the internal exons 2–28 of APOB. [file 1471-2199-14-5-S1.pdf]

	3'SS				*5'SS*				*3'+5'SS*	
**Exon**	**Motif**	**Start**	**Sequence**	**Score**	**Motif**	**End**	**Sequence**	**Score**	**Score**	**Length**
1					209	210	CG|GTGAGT	88.5		
2	509	523	CTCCTGTCTTACAG|A	90.1	560	561	AA|GTAAGG	83	173.1	39
3	1584	1598	TCTCCCCACGTTAG|A	78.8	1712	1713	AG|GTATGG	84.1	162.9	116
4	2977	2991	CCCTGAATTCTCAG|G	85.8	3135	3136	AG|GTAAGT	100	185.8	146
5	5949	5963	TCTCTCTGGGACAG|G	87.4	6115	6116	TG|GTGAGG	84.3	171.7	154
6	6805	6819	TTCTTCCTCCATAG|G	87.8	6973	6974	TG|GTAAGT	92.2	180	156
7	8352	8366	AATCTCTTTCATAG|A	82.5	8489	8490	AA|GTAGGT	76.8	159.3	125
8	9159	9173	TCCTTGGTTACCAG|G	89.2	9257	9258	AG|GTAAGA	94.4	183.6	86
9	10542	10556	TTTCCATCTTCCAG|G	96.3	10774	10775	AG|GTATCT	74.5	170.8	220
10	11479	11493	GTCTTTCTTCTCAG|C	91.6	11719	11720	AA|GTGAGT	84.3	175.9	228
11	14045	14059	CTCTTATTCTGTAG|C	80.6	14175	14176	GG|GTAATC	71.9	152.5	118
12	14275	14289	TTTCTTCAATGCAG|G	96.7	14434	14435	AG|GTAAAG	81	177.7	147
13	15522	15536	TCTTTTGACTGCAG|G	96.7	15746	15747	GA|GTAAGT	79.8	176.5	212
14	15995	16009	TTCTTCTTATCTAG|T	81.7	16245	16246	AG|GTAAGT	100	181.7	238
15	17096	17110	ATTTTCTTTCTCAG|A	94.9	17285	17286	AG|GTGTGT	85.4	180.3	177
16	18936	18950	TACTAAACTCTCAG|G	84.2	19140	19141	TG|GTAAGT	92.2	176.4	192
17	20368	20382	TGTTGGGTCTACAG|A	84.3	20548	20549	AC|GTAAGA	80.8	165.1	168
18	21018	21032	TTTCTGACTTATAG|A	85.3	21242	21243	GG|GTAATT	77.7	163	212
19	24155	24169	TTGTTCTTCACCAG|C	89.4	24350	24351	AG|GTTAGA	84.3	173.7	183
20	24947	24961	TGTAATGTCTTTAG|A	74.7	25081	25082	AG|GTGAGT	96.7	171.4	122
21	27411	27425	ATACACTCGTCCAG|G	82.2	27634	27635	AG|GTAAAG	81	163.2	211
22	28515	28529	TTTCTTTACCATAG|T	81.7	28703	28704	TG|GTATGT	80.8	162.5	176
23	28800	28814	CTACGTATTTTCAG|A	84.3	29000	29001	TT|GTAAGT	79.4	163.7	188
24	29467	29481	CTGTCATTTTGTAG|G	85.9	29625	29626	AG|GTAAAA	79.9	165.8	146
25	30527	30541	GTTGTTCATTTCAG|C	88.3	30913	30914	AG|GTGAGC	90.9	179.2	374
26	31409	31423	CATTTTTATGTTAG|G	83.2	38993	38994	TG|GTAAGA	86.5	169.7	7572
27	39385	39399	TCTCTCCTATACAG|T	88.6	39512	39513	CA|GTATGG	63.5	152.1	115
28	39608	39622	TTTTCTGCTTTCAG|G	99.1	39804	39805	AG|GTAAAT	85.6	184.7	184
29	40726	40740	TTCTCTCTGTTTAG|T	81.4						
**Min**				74.7				63.5	152.1	39
**Max**				99.1				100	185.8	374
