# Supplementary material for: Multiple tandem splicing silencer elements suppress aberrant splicing within the long exon 26 of the human Apolipoprotein B gene
Source: BMC Mol Biol. 2013 Feb 7;14:5. doi: 10.1186/1471-2199-14-5 (PMC3640928; doi:10.1186/1471-2199-14-5)
Supplement: Additional file 2: Table S2 — Maximum Entropy Modelling scores [19] for 3′ and 5’ splice sites. Only motifs with higher scores than the native splice sites (italics) shown. Branch point sequences motifs with appropriate strength (score >70) and positioned within 50 nucleotides of a 3′ splice site capable of generating a pseudoexon [20]. [file 1471-2199-14-5-S2.pdf]

Position	BPS		3′ splice sites		5′ splice sites	
	sequence	score	sequence	score	sequence	score
1			*TCTTTTCATTTTTATGTTAG |GAT*	*7.68*		
4855	TGCTCAT	90				
4905			AAATTCTCTTTTCTTTTCAG | CCC	8.71		
5048					CAA | GT AAGT	10.08
5739			TCTCAAGCTTTCTCTTCCAG | ATT	8.41		
6719			TTCATTCTGGGTCTTTCCAG | AGC	8.34		
7047			GTTCCATGTCCCATTTACAG | ATC	10.04		
7428			TGTCATTCCTTCCTTTCAAG | CAC	7.73		
7572					*ATG | GT AAGA*	*9.48*
