# Supplementary figures and images for: Multiple tandem splicing silencer elements suppress aberrant splicing within the long exon 26 of the human Apolipoprotein B gene
Source: BMC Mol Biol. 2013 Feb 7;14:5. doi: 10.1186/1471-2199-14-5 (PMC3640928; doi:10.1186/1471-2199-14-5)

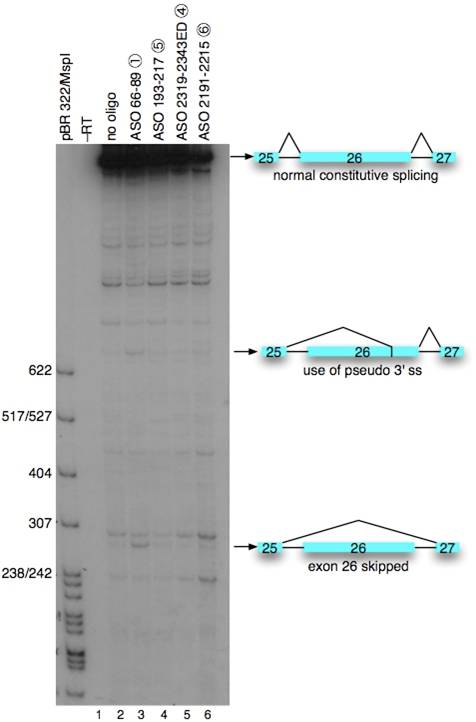

Supplement: Additional file 3: Figure S1 — Antisense oligonucleotides directed against identified ESS elements of APOB exon 26 in HepG2 cells – additional control oligonucleotide included. In lanes 1 and 2 the cells were not transfected with ASOs. The –RT control PCR (lane 1) was performed without reverse transcriptase. Lanes 3–6 were transfected with the indicated ASOs at 250 nM each. An additional control oligonucleotide targeting a neutral sequence is shown in lane 6. Cells were incubated for 48 hours and RT-PCR was carried out on the total RNA extracted from these cells with oligonucleotides annealing to the adjacent exons 25 and 27. The positions of the bands corresponding to the APOB mRNA species with constitutive exon 26 inclusion (top), pseudo 3′ splice site activation (middle) and skipping of exon 26 (bottom) are indicated on the right side. Fragment lengths in bp of the markers are indicated on the left side. [file 1471-2199-14-5-S3.jpeg]

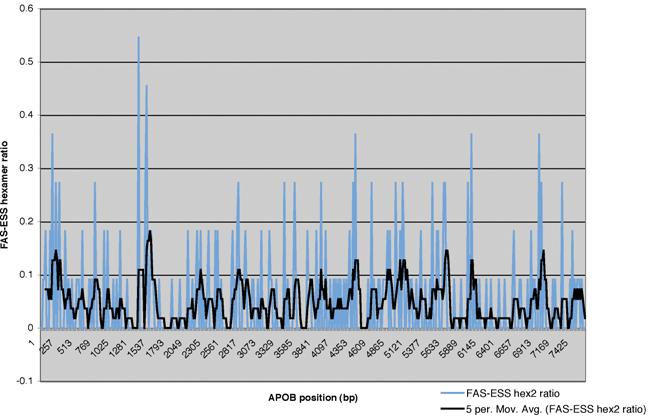

Supplement: Additional file 4: Figure S2 — Frequency of FAS-ESS hex2 hexamers in the APOB exon 26 sequence. FAS-ESS hex2 subset refers to those hexamers found at least twice within recovered decamers from the FAS-ESS procedure [47]. Hexamers were counted in a non-overlapping 16 bp windows and normalised to the number of possible hexamers per 16 nt. Blue line shows normalised hexamer ratio plotted for each window. Black line shows a moving average over 5 windows. [file 1471-2199-14-5-S4.jpeg]
