# Supplementary material for: Multiple tandem splicing silencer elements suppress aberrant splicing within the long exon 26 of the human Apolipoprotein B gene
Source: BMC Mol Biol. 2013 Feb 7;14:5. doi: 10.1186/1471-2199-14-5 (PMC3640928; doi:10.1186/1471-2199-14-5)
Supplement: Additional file 5: Figure S3 — Computational identification of potential ESS sequences within the exon 26 sequence. The exon 26 sequence is shown in black, with numbers above denoting the position in exon 26. 25-mers tested in the DNA ligase III reporter system are shown below the exon 26 sequence, sequences with ESS activity are denoted by white-on-black text, neutral sequences are denoted by black-on-white text. Sequences containing hexamers identified by the FAS-ESS server program are in bold [47]. Matches to the PESS sequences identified by Zhang and Chasin [8] are underlined. [file 1471-2199-14-5-S5.pdf]

```
|1       |10       |20       |30       |40       |50       |60       |70       |80       |90       |100
GAUCUGGAGAAACAACAUAUGACCACAAGAAUACGUUCACACUAUCAUGUGAUGGGUCUCUACGCCACAAAUUUCUAGAUUCGAAUAUCAAAUUCAGUCA
<---- 1-25:Neutral  ---->                       <---- 49-73:Neutral ---->                       <---
                <---- 17-41:Neutral ---->                       <------ 65-89:ESS ------>
                                <---- 33-57:Neutral ---->                       <------ 81-105:ESS -

|101     |110      |120      |130      |140      |150      |160      |170      |180      |190      |200
UGUAGAAAAACUUGGAAACAACCCAGUCUCAAAAGGUUUACUAAUAUUCGAUGCAUCUAGUUCCUGGGGACCACAGAUGUCUGCUUCAGUUCAUUUGGAC
--- 97-121:ESS ----->                       <--- 145-169:Neutral --->                       <-------
            <--- 113-137:Neutral --->                       <--- 161-185:Neutral --->
---->                       <----- 129-153:ESS ----->                       <--- 177-201:Neutral ---

|201     |210      |220      |230      |240      |250      |260      |270      |280      |290      |300
UCCAAAAAGAAACAGCAUUUGUUUGUGAAACAGCAUUUGUUUGUCAAAGAAGUCAAGAUUGAUGGGCAGUUCAGAGUCUCUUCGUUCUAUGCUAAAGGCA
193-217:Neutral->                       <--- 241-265:Neutral --->
        <--- 209-233:Neutral --->                       <----- 257-281:ESS ----->
>                       <--- 225-249:Neutral --->
```
