# Supplementary material for: Multiple tandem splicing silencer elements suppress aberrant splicing within the long exon 26 of the human Apolipoprotein B gene
Source: BMC Mol Biol. 2013 Feb 7;14:5. doi: 10.1186/1471-2199-14-5 (PMC3640928; doi:10.1186/1471-2199-14-5)
Supplement: Additional file 6: Figure S4 — Computational identification of potential ESS sequences within the exon 26 sequence derived from fragment 6. The exon 26 sequence is shown in black, with numbers above denoting the position in exon 26. 25-mers tested in the DNA ligase III reporter system are shown below the exon 26 sequence, sequences with ESS activity are denoted by white-on-black text, neutral sequences are denoted by black-on-white text. Sequences containing hexamers identified by the FAS-ESS server program are in bold [47]. Matches to the PESS sequences identified by Zhang and Chasin [8] are underlined. [file 1471-2199-14-5-S6.pdf]

|2140 |2150 |2160 |2170 |2180 |2190 |2200 |2210 |2220 |2230

CACAGCAAGCUAAUGAUUAUCUGAAUUCAUUCAAU**UGGGAG**AGAC**AAGUUUC**ACAUG**CCAAGG**AGAAACUGACUGCUCUCACAAAAA**AGUAUAG**AAUUAC

<---- 2143-2167:ESS ----> <-- 2191-2215:Neutral --> <

<---- 2159-2183:ESS ----> <---- 2207-2231:ESS ---->

<-- 2175-2199:Neutral --> <----- 2223-2247:

|2240 |2250 |2260 |2270 |2280 |2290 |2300 |2310 |2320 |2330

AGAAAAUGAUAUACAAAUUGCA**UUAGAU**GAUGCCAAAAUCAACUUUAAUGAAAAACUAUCCAACUGCAGACAUAUAUGAUACAAUUUGAUC**AGUAUA**UUU

---- 2239-2263:ESS ----> <-- 2287-2311:Neutral --> <----

<---- 2255-2279:ESS ----> <---- 2303-2327:ESS ---->

ESS ---> <---- 2271-2295:ESS ----> <--- 2319-2343 ED:ESS

|2340 |2350 |2360 |2370 |2380 |2390 |2400 |2410 |2420

AAAG**AUAGUUA**UGAUUUACAUGAUUUGAAAAUAGCUAUUGCUAAUAUUAUUGAUGAAAUCAUUGAAAAAUUAAAAAGUCUU

2335-2359:ESS ----> <---- 2383-2407:ESS ---->

<---- 2351-2375:ESS ---->

---> <---- 2367-2391:ESS ---->
