# Supplementary material for: Multiple tandem splicing silencer elements suppress aberrant splicing within the long exon 26 of the human Apolipoprotein B gene
Source: BMC Mol Biol. 2013 Feb 7;14:5. doi: 10.1186/1471-2199-14-5 (PMC3640928; doi:10.1186/1471-2199-14-5)
Supplement: Additional file 7: Table S3 — Degree of base-pairing secondary structure predicted for regions of APOB exon 26 using the Mfold web server [35]. These include the native 3′ and 5′ splice sites, parts of region A1 (1–281) and A6 (2143–2407) investigated in vitro (see Figures 2 & 3) and the regions of exon 26 targeted by the ASOs 1–6. Secondary structure was analysed using the whole of exon 26 in addition to the entire length of the adjacent introns either side. Figures shown are the number of nucleotides predicted to be base-paired using the 4 most stable structures predicted (range of ΔG −2245.70 to −2244.2 kcal/mol) Final column gives the average number of nucleotides base-paired and the %nucleotides base-paired for the given sequences. [file 1471-2199-14-5-S7.doc]

|  |  | **Base-pairing** | | | | | |
| --- | --- | --- | --- | --- | --- | --- | --- |
| **Region** | **sequence** | **length** | **1** | **2** | **3** | **4** | **Average** |
| ΔG/ kcal/mol | | | -2245.70 | -2245.6 | -2245.5 | -2244.2 |  |
| 3′ splice site (native) | -10-10 | 20 | 14 | 14 | 14 | 14 | 14 (70.0%) |
| 5′ splice site (native) | 5262 -5583 | 20 | 10 | 10 | 13 | 10 | 10.8 (53.8%) |
| A1 region | 1-281 | 281 | 161 | 167 | 166 | 169 | 165.8 (59.0%) |
| ASO 1 target | 64-89 | 25 | 18 | 14 | 17 | 18 | 16.8 (67.2%) |
| ASO 2 target | 97-121 | 25 | 11 | 6 | 11 | 11 | 9.8 (39.2%) |
| ASO 3 target | 129-153 | 25 | 11 | 13 | 12 | 11 | 11.8 (59.0%) |
| ASO 5 (neutral) target | 193-217 | 25 | 16 | 19 | 13 | 16 | 16 (47.2%) |
| A6 region | 2143-2407 | 265 | 167 | 168 | 179 | 182 | 174 (65.7%) |
| ASO 4 (ED) target | 2319-2343 | 25 | 19 | 19 | 19 | 19 | 19 (76.0%) |
| ASO 6 (neutral) target | 2191-2215 | 25 | 8 | 8 | 8 | 8 | 8 (32.0%) |
